# Nerve ultrasound in hereditary transthyretin amyloidosis: red flags and possible progression biomarkers

**DOI:** 10.1007/s00415-020-10127-8

**Published:** 2020-08-04

**Authors:** Alessandro Salvalaggio, Daniele Coraci, Mario Cacciavillani, Laura Obici, Anna Mazzeo, Marco Luigetti, Francesca Pastorelli, Marina Grandis, Tiziana Cavallaro, Giulia Bisogni, Alessandro Lozza, Chiara Gemelli, Luca Gentile, Mario Ermani, Gian Maria Fabrizi, Rosaria Plasmati, Marta Campagnolo, Francesca Castellani, Roberto Gasparotti, Carlo Martinoli, Luca Padua, Chiara Briani

**Affiliations:** 1 Department of Neurosciences, University of Padova, Via Giustiniani 5, 35128 Padova, Italy; 2 Padova Neuroscience Center (PNC), University of Padova, Padova, Italy; 3 Neuroriabilitazione Ad Alta Intensità, Fondazione Policlinico Universitario A. Gemelli IRCCS, Rome, Italy; 4 CEMES-EMG Lab, Synlab Group, Padova, Italy; 5 Amyloidosis Research and Treatment Centre, Fondazione IRCCS Policlinico San Matteo, Pavia, Italy; 6 Unit of Neurology and Neuromuscular Diseases, Department of Clinical and Experimental Medicine, University of Messina, Messina, Italy; 7 Neurology Unit, Fondazione Policlinico Universitario Gemelli IRCCS, Rome, Italy; 8 IRCSS Istituto Scienze Neurologiche Città Di Bologna, Bologna, Italy; 9 Department of Neuroscience, Rehabilitation, Ophthalmology, Genetics, Maternal and Child Health (DiNOGMI), University of Genova, Genova, Italy; 10 Ospedale Policlinico San Martino IRCCS, Genova, Italy; 11 Neurology Unit, Department of Neuroscience, Biomedicine and Movement Sciences, University of Verona, Verona, Italy; 12 Centro Clinico NEMO Adulti, Roma, Italy; 13 Department of Medical and Surgical Specialties, Radiological Sciences, and Public Health, University of Brescia, Brescia, Italy; 14 Department of Scienze Della Salute, University of Genova, Genova, Italy; 15 Department of Geriatrics, Neurosciences and Orthopaedics, Catholic University of the Sacred Heart, Rome, Italy

**Keywords:** Transthyretin amyloidosis, Nerve ultrasound, Carpal tunnel syndrome, ATTRv, Amyloidotic polyneuropathy

## Abstract

**Background:**

Diagnostic delay of hereditary transthyretin amyloidosis (ATTRv, v for variant) prevents timely treatment and, therefore, concurs to the mortality of the disease. The aim of the present study was to explore with nerve ultrasound (US) possible red flags for early diagnosis in ATTRv patients with carpal tunnel syndrome (CTS) and/or polyneuropathy and in pre-symptomatic carriers.

**Methods:**

Patients and pre-symptomatic carriers with a TTR gene mutation were enrolled from seven Italian centers. Severity of CTS was assessed with neurophysiology and clinical evaluation. Median nerve cross-section area (CSA) was measured with US in ATTRv carriers with CTS (TTR-CTS). One thousand one hundred ninety-six idiopathic CTS were used as controls. Nerve US was also performed in several nerve trunks (median, ulnar, radial, brachial plexi, tibial, peroneal, sciatic, sural) in ATTRv patients with polyneuropathy and in pre-symptomatic carriers.

**Results:**

Sixty-two subjects (34 men, 28 women, mean age 59.8 years ± 12) with TTR gene mutation were recruited. With regard to CTS, while in idiopathic CTS there was a direct correlation between CTS severity and median nerve CSA (*r* = 0.55, *p* < 0.01), in the subgroup of TTR-CTS subjects (16 subjects, 5 with bilateral CTS) CSA did not significantly correlate with CTS severity (*r* = − 0.473). ATTRv patients with polyneuropathy showed larger CSA than pre-symptomatic carriers in several nerve sites, more pronounced at brachial plexi (*p* < 0.001).

**Conclusions:**

The present study identifies nerve morphological US patterns that may help in the early diagnosis (morpho-functional dissociation of median nerve in CTS) and monitoring of pre-symptomatic TTR carriers (larger nerve CSA at proximal nerve sites, especially at brachial plexi).

## Introduction

The most common neurological manifestations of hereditary transthyretin amyloidosis (ATTRv, v for variant) in non-endemic regions, where misdiagnosis is still a considerable burden, are a length-dependent axonal sensorimotor polyneuropathy and carpal tunnel syndrome (CTS), more often bilateral [1, 2]. Onset of CTS usually occurs several years before systemic symptoms develop and it has therefore been advocated as a potentially red flag [3, 4]. However, idiopathic CTS is very common (10% of prevalence) thus making difficult an early diagnosis of ATTRv in sporadic patients in the presence of only CTS [5, 6]. Moreover, the accurate monitoring of pre-symptomatic mutation carriers is crucial for detecting early signs of disease and starting timely the proper therapy. Indeed, recently new therapies are available for the treatment of ATTRv [7–9] making an early and correct diagnosis critical for the care of these patients. In idiopathic CTS, neurophysiological and US findings generally correlate, that is the more severe the CTS, the larger the median nerve cross-sectional area (CSA) evaluated by ultrasound (US) at wrist [10]. Interestingly, the only two studies focusing on nerve US in ATTR patients failed to detect such correlation between neurophysiological and US findings in CTS [11], but reported CSA enlargement in entrapment sites and proximal nerves at upper limbs [12]. Both studies however did not include ATTRv subjects with isolated CTS making therefore difficult to dissect the contribution of the coexisting axonal polyneuropathy on nerve findings.

In the present study we aimed at identifying a possible morphological US pattern able to distinguish idiopathic CTS from ATTRv-associated CTS. Moreover, we performed an extensive nerve US evaluation in both ATTRv patients with polyneuropathy and pre-symptomatic carriers to identify possible biomarkers for early diagnosis. Finally, difference of US findings among different TTR gene mutations was assessed.

## Patients and method

### Patient enrollment

Both ATTRv patients and pre-symptomatic carriers with mutated TTR gene (known pathological mutation) aged > 18 years were recruited from seven Italian centers. Subject with diabetes mellitus or other conditions possible cause of neuropathy (such as previous chemotherapy) were excluded. Enrollment period run from March 2016 to December 2018. A control group of 1196 subjects with idiopathic CTS was enrolled for sub-analyses from the dataset of subjects evaluated in Padova (CEMES-EMG Lab, SYNLAB, Padova, Italy): all the patients had symptoms/signs of CTS in absence of signs or symptoms or family history of polyneuropathy, autonomic dysfunction or cardiomyopathy. For the first aim of the study (evaluation of CTS) we selected ATTRv mutation carriers with isolated CTS (no polyneuropathy or cardiomyopathy were present, as from a detailed neurological and cardiologic clinical and instrumental evaluation). This group was named TTR-CTS and was compared to the control group of idiopathic CTS. For the second aim of the study (extended US evaluation of peripheral nervous system) we compared ATTRv patients with polyneuropathy to pre-symptomatic subjects (TTR mutation carriers with no polyneuropathy and cardiomyopathy, or other ATTRv manifestations except for CTS). The latter group included subjects without CTS, subjects with symptomatic, untreated CTS at the time of evaluation and subjects previously surgically treated for CTS.

### Neurological evaluation

All the subjects underwent a comprehensive neurological evaluation. The polyneuropathy was scored with the NIS-LL (Neuropathy Impairment Score—Lower Limbs) scale [13]. Presence and severity of CTS were also assessed with *Hi-Ob* scale [14].

### Neurophysiological study

Neurophysiological studies were performed according to the guidelines of the American Association of Electrodiagnostic Medicine, American Academy of Neurology, and the American Academy of Physical Medicine and Rehabilitation [15]. In detail, sensory nerve action potential (SNAP) amplitude and sensory conduction velocities (SCV) of ulnar, radial and sural nerves, compound motor action potential (cMAP) and motor conduction velocities (MCV) of ulnar and peroneal nerve were performed on the non-dominant side. cMAPs were evoked from the median nerve bilaterally (stimulating at wrist and elbow, recording at the abductor pollicis brevis). Distal motor latencies (DML), conduction velocity and presence of conduction blocks were ascertained at and outside the compression sites. The skin temperature was maintained at ≥ 32 °C throughout the study. Polyneuropathy was defined according to accepted criteria [16].

For the assessment of possible CTS, the following neurophysiological studies of the median nerve were performed bilaterally: (1) SCV in two digit/wrist segments (the first and the third digit), and (2) DML from the wrist to the thenar eminence. Moreover, when the standard tests yield normal results, segmental (over a short distance of 7 to 8 cm) or comparative studies (e.g., median/ulnar comparison or distal–proximal ratio) were performed.

According to Padua’s Scale, severity of CTS was classified into five classes [17] and was considered "negative CTS" if normal findings were present in all tests (including comparative or segmental tests): (1) "minimum CTS ", pathological findings only on segmental or comparative test; (2) "mild CTS", slowing of SNCV the median nerve (finger—wrist segment) and the normality of the DML; (3) "moderate CTS ", slowing of sensory conduction of the median finger—wrist segment and abnormal DML; (4) "severe CTS", absence of at least one sensory response and abnormal DML; 5) "extreme CTS", absence of motor and sensory response.

### Ultrasound evaluation

US evaluation was performed as previously described [18] by three neurophysiologists (DC, MC, CM) with expertise in nerve ultrasound with a US system equipped with high frequency linear transducer, frequency range 10–18 MHz (MyLab Seven Esaote, Genova, Italy and Toshiba Aplio 400). The best visualized cross-sectional area (CSA) was measured with the ‘‘ellipse method’’ when applicable or the ‘ ‘tracing method’’ when the nerve had an irregular shape. The mean CSA value of three measurements was considered. The course of median and ulnar nerves was followed bilaterally from axilla to wrist; measurement of nerve CSA at wrist, forearm, and arm were performed. Ulnar nerve CSA was measured also at the elbow (see below). The course of peroneal nerve was followed bilaterally from the popliteal fossa to the proximal third of the leg with measurement of the nerve CSA at popliteal fossa. Brachial plexus was measured at supraclavicular space at the level of divisions, after the trunks and before the cords. The course of tibial nerve was followed bilaterally in the popliteal fossa with measurement of the nerve CSA. The course of sural nerve was followed bilaterally from the median third of the leg to the malleolus with measurement of the nerve CSA at the median third of the leg. The following nerve trunks were evaluated bilaterally: median nerve at wrist, forearm, elbow, arm and axilla; ulnar nerve at wrist, forearm, elbow, arm and axilla; posterior interosseous nerve at forearm; radial nerve at spiral groove; fibular nerve at fibular head and popliteal fossa; tibial nerve at the ankle and popliteal fossa; sciatic nerve at proximal thigh; sural nerve at the distal calf; brachial plexus at supraclavicular space; C5, C6 and C7 roots after leaving transversal processes.

### Idiopathic carpal tunnel syndrome

Beside the evaluation of TTR subjects, data collected from the evaluation of subjects affected with CTS performed in the last 5 years by an experienced neurophysiologist (MC) were analyzed. Entity of CTS was classified according to Padua’s Scale [17] and US median nerve CSA at wrist was measured in the same way as described above for TTR subjects.

### Statistical analysis

Normality was tested with Kolmogorov–Smirnov method and variance equality with Levine test. Group comparison was performed with Mann–Whitney test for ordinal variables and student *T* test for normal distributed variables in independent groups. Linear correlation between two variables was assessed with Spearman rho when at least one of the two variables was ordinal and Pearson *r* when both the variables presented with a normal distribution. Significant level was set at *p* < 0.05. Bonferroni correction was applied for multiple comparisons. IBM SPSS Statistic version 23 was used for statistical analyses.

### Standard protocol approvals, registrations, and patient consents

Enrolled subjects gave written informed consent to participate to the study, which was approved by the local ethical committees of all the involved centers. The study has been performed in accordance with the ethical standards laid down in the 1964 Declaration of Helsinki and its later amendments and national laws have been observed.

## Results

### Subjects

Sixty-two subjects (34 men, 28 women) with a pathological TTR gene mutation were enrolled. Mean age was 59.8 years (± 12, range 36–86), mean BMI was 25.0 (± 5, range 14–38). The most frequent mutations were Phe64Leu (22 subjects), Val30Met (13 subjects), Glu89Gln (11) subjects (see Table 1). According to the neurophysiologic findings, 34 (55%) ATTRv subjects (mean age 64.4, range 36–86, mean BMI 24.2, range 14–38) were diagnosed with axonal polyneuropathy, 28 subjects (45%) did not show symptoms or signs of systemic amyloidosis including polyneuropathy (based on clinical and neurophysiological evaluation) or cardiomyopathy and were considered as pre-symptomatic *carriers* (mean age 54.0, mean BMI 26.0). Mean NIS-LL (range 0–88) value among ATTRv patients with polyneuropathy was 28.9 (range 5–67). Prevalence of ATTRv polyneuropathy across the different mutations is reported in Table 2.

**Table 1:** TTR gene mutations in our ATTRv populations

Mutation	Number of subjects	Mean age (yrs)
Phe64Leu	22	64.3
Val30Met	13	62.3
Glu89Gln	11	53.1
Ile68Leu	5	64.0
Thr49Ala	4	45.5
Tyr78Phe	2	72.0
Ala120Ser	1	62.0
Ala36Pro	1	48.0
Arg34Thr	1	41.0
Glu62Lys	1	60.0
Gly47Ala	1	39.0
Total	62	60.0

**Table 2:** Prevalence and severity of ATTRv polyneuropathy (PN) in the different genotypes

Mutation	Number of subjects with PN	NIS-LL (mean) in subjects with PN
Phe64Leu	8/22 (36%)	32.4
Val30Met	10/13 (77%)	41.2
Glu89Gln	7/11 (64%)	20.1
Ile68Leu	2/5 (40%)	6.5
Thr49Ala	4/4 (100%)	25.0
Others	3/7 (43%)	19.6
Total	34 (55%)	28.9

*NIS-LL* Neuropathy Impairment Score at Lower Limbs, *PN* polyneuropathy

### Ultrasound of median nerve at wrist in patients with idiopathic carpal tunnel syndrome

**Fig. 1:**
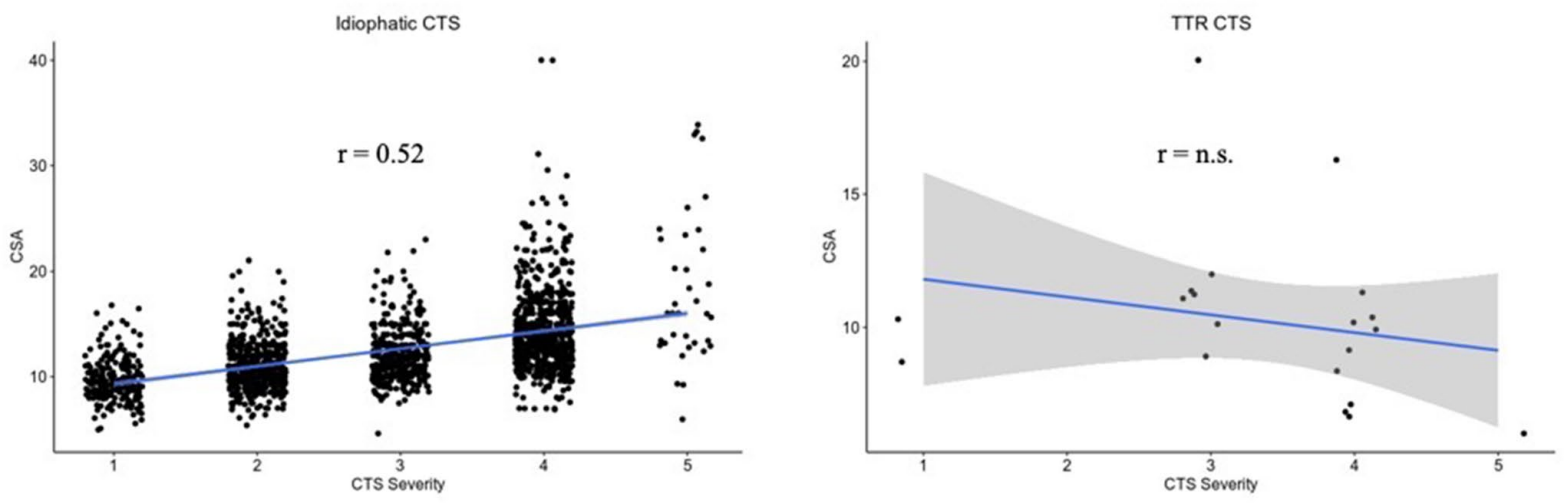
Correlation between severity and cross-sectional area in idiopathic CTS and ATTRv carriers. In idiopathic CTS (**a**) there is direct and significant correlation between severity (x axis) and CSA of median nerve at wrist (y axis). Conversely, in subject with ATTRv carriers (**b**), there was a non-significant tendency to a reverse correlation*. CTS* carpal tunnel syndrome, ATTRv: hereditary Transthyretin amyloidosis, *TTR* Transthyretin, *CSA* cross-section area, *n.s.* not significant

Data from 1196 subjects with CTS were collected. Among them, 473 had bilateral CTS, therefore a total of 1669 hands with CTS was evaluated. Mean age was 57.5 years (range 20–94), 392 were men and 1277 women, mean BMI was 26.4 (range 16.2–46.7). There were 913 right and 756 left CTS. According to Padua’s Scale^16^, 210 CTS were classified as grade 1, 462 as grade 2, 348 as grade 3, 614 as grade 4, and 35 as grade 5. Mean CSA of median nerve at wrist was 12.5 ± 3.7 (range 4.6–40). A correlation between CSA and severity (expressed with Padua’s scale) emerged when the whole group was considered (Pearson *r* = 0.55, *p* < 0.01) (Fig. 1a). When considering only subjects older than 65 years (498 hands), the mean median nerve CSA at wrist was 12.9 ± 3.8. Correlation between CSA and Padua’s scale was 0.49 (*p* < 0.01). When considering only subjects aged ≤ 65 years (1171 hands), mean CSA was 12.3 ± 3.7. Correlation between median nerve CSA at wrist and Padua’s scale was 0.52 (*p* < 0.01). Therefore, in idiopathic CTS, CTS severity and median nerve CSA at wrist directly correlate, regardless of age (Fig. 1a).

### Ultrasound of median nerve at wrist and carpal tunnel syndrome in ATTRv mutation carriers with isolated CTS (TTR-CTS)

Among the enrolled subjects, 49/62 (79%) presented with CTS at the time of evaluation (or had previously undergone surgery for CTS) in at least one hand. In 22 subjects (35%) CTS was the first manifestation of the disease (isolated or concomitant with other symptom/signs). For pre-symptomatic individuals, only untreated hands with CTS were included in the analyses. Since the aim of our study was to find a potential marker able to distinguish idiopathic CTS from TTR-related CTS, we focused on pre-symptomatic carriers with only CTS (still not with polyneuropathy), therefore 21 hands (11 right, 10 left) from 16 patients (10 women, 6 men; 5 patients had bilateral CTS; mean age 53.9 ± 12.2, mean BMI 24.4 ± 4) were considered in this sub-analysis. ATTRv subjects with polyneuropathy were excluded from this sub-analysis because it would have been difficult to understand whether the findings at median nerve were due to the CTS itself or to the established polyneuropathy. With regard to the CTS severity [16], 2 hands had a CTS grade 1, 8 hands had grade 3, 10 hands grade 4 and 1 hand had grade 5.

Mean CSA of median nerve at wrist was 10.0 ± 3.2 (range 6–20). No correlation between CSA and CTS severity emerged (Fig. 1b).

### Comparison between idiopathic and TTR-CTS

**Fig. 2:**
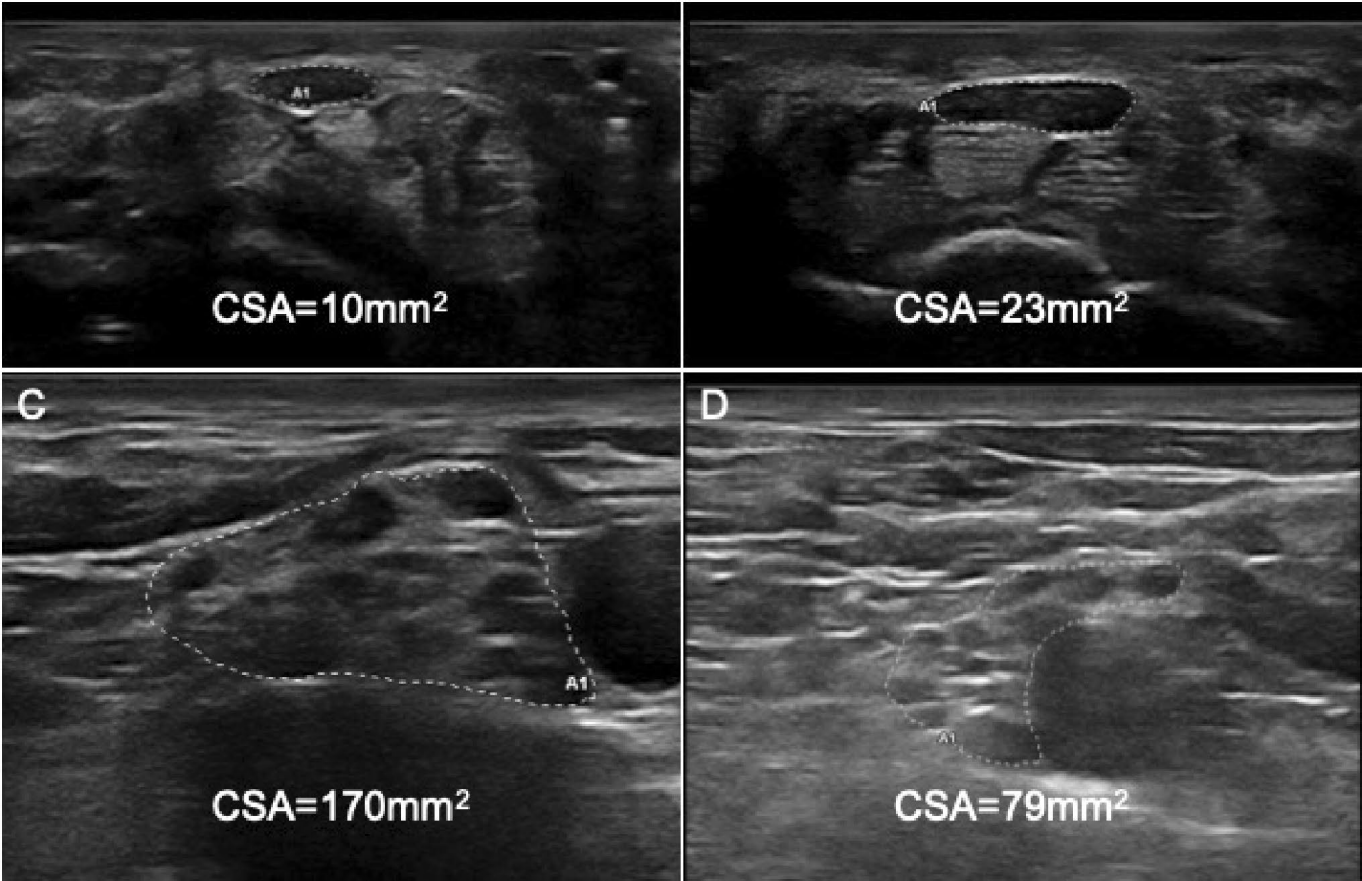
Nerve ultrasound of median nerve at wrist and brachial plexus in supraclavicular space. Panel **a** shows a severe CTS (grade 4, accordingly to Padua’ scale) in an ATTRv woman, CSA was within normal limit (10mm^2^), while in **b** an idiopathic CTS of the same severity showed a markedly enlarged CSA (23 mm^2^). In C and D right brachial plexus at supraclavicular space are showed. In **c** is reported the enlarged plexus of an ATTRv patient (with late-onset Val30Met mutation) with polyneuropathy, in **d** the normal plexus of the pre-symptomatic son of the same patients. *CSA* Cross sectional area

Gender, side of CTS, BMI and age did not significantly differ between patients with idiopathic CTS and patients with TTR-CTS. CSA was significantly (*p* = 0.003) lower in TTR-CTS group (Fig. 2a–b). When calculating the ROC curve, the area under the curve (AUC) for CSA was 0.74 (0.63–0.85 95% confidence interval). Greater Youden index was for CSA = 10.0 (sensitivity 0.76, specificity 0.67).

### Nerve ultrasound of peripheral nervous system in ATTRv patients with polyneuropathy (ATTRv-PN) and carriers

ATTRv-PN patients showed statistically significant larger nerve CSA than pre-symptomatic carriers in several sites (Table 3).

**Table 3:** Nerve Ultrasound CSA in ATTRv patients with polyneuropathy (PN) and pre-symptomatic carriers

Site	Mean SD (mm^2^)		*p* values
	ATTRv-PN (*N* = 34)	ATTRv carriers (*N* = 28)	
Median nerve at wrist	9.7 (2.4) *n* = 67	8.8 (2.7) *n* = 55	0.033
Median nerve at forearm	6.7 (1.9) *n* = 67	5.8 (1.5) *n* = 56	0.008
Median nerve at elbow	10.2 (3.0) *n* = 67	8.2 (1.9) *n* = 56	0.000*
Median nerve at arm	10.4 (3.3) *n* = 67	8.3 (2.1) *n* = 56	0.000*
Median nerve at axilla	11.1 (4.6) *n* = 49	8.5 (1.9) *n* = 56	0.000*
Ulnar nerve at wrist	5.1 (1.4) *n* = 67	5.0 (2.0) *n* = 56	0.705
Ulnar nerve at forearm	5.9 (1.6) *n* = 67	5.5 (1.5) *n* = 56	0.262
Ulnar nerve at elbow	9.3 (2.5) *n* = 67	7.8 (2.1) *n* = 56	0.000*
Ulnar nerve at arm	7.7 (2.3) *n* = 67	6.2 (1.5) *n* = 56	0.000*
Ulnar nerve at axilla	7.1 (2.0) *n* = 49	6.2 (1.5) *n* = 56	0.014
Radial nerve at spiral groove	5.5 (2.0) *n* = 65	4.8 (1.7) *n* = 56	0.034
Posterior interosseous nerve	2.1 (0.7) *n* = 60	1.9 (0.7) *n* = 56	0.109
Brachial plexus at supraclavicular space	97.5 (25.2)* n* = 52	65.5 (16.2) *n* = 46	0.000*
C5 root	6.9 (2.3) *n* = 55	5.9 (1.5) *n* = 54	0.007
C6 root	9.7 (2.5) *n* = 55	7.8 (2.2) *n* = 54	0.000*
C7 root	10.6 (3.1) *n* = 51	8.9 (1.9) *n* = 53	0.001*
Fibular nerve at fibular head	9.4 (2.8) *n* = 65	8.0 (2.3) *n* = 56	0.003
Fibular nerve at popliteal fossa	7.9 (2.7) *n* = 65	6.6 (1.8) *n* = 56	0.002*
Tibial nerve at tarsal tunnel	10.2 (3.6) *n* = 60	9.5 (1.9) *n* = 52	0.185
Tibial nerve at popliteal fossa	25.1 (6.2) *n* = 61	23.3 (4.8) *n* = 52	0.083
Sural nerve	2.4 (0.6) *n* = 33	2.2 (0.7) *n* = 28	0.369
Sciatic nerve at mid-thigh	42.7 (10.1) *n* = 26	35.9 (7.6) *n* = 28	0.008

*N* patients for each group, *n*  number of CSA measurements for each site (right and left), * significant after multiple comparisons correction

At brachial plexus CSA of ATTRv-PN patients was 97.5 mm^2^ (SD 25.2), while CSA of pre-symptomatic carries was 65.5 mm^2^ (*p* < 0.001) (SD 16.2) (Fig. 2c–d). Since at brachial plexus the difference between the two groups was measurable in terms of dozens of mm^3^, a ROC curve analysis was performed at this site demonstrating an AUC value of 0.869 (0.799–0.938).

Among ATTRv-PN patients, nerve CSA significantly correlated with NIS-LL score (after Bonferroni correction) when measured in the following sites: median nerve at axilla (*r* = 0.59), ulnar nerve at arm (*r* = 0.53), radial nerve at spiral groove (*r* = 0.58). Nerve CSA did not correlate with age in any of the explored sites.

Analysis of US data in ATTRv-PN patients across the most frequent mutations showed that at brachial plexus the nerve enlargement was much larger in patients carrying the Val30Met and Glu89Gln mutations (Table 4).

**Table 4:** Nerve ultrasound CSA in TTR-PN patients among the different mutations (mutations with ≤ 2 subjects with polyneuropathy are not included)

Site	Mean and standard deviation (mm^3^) of nerve CSA			
	Phe64Leu	Val30Met	Glu89Gln	Thr49Ala
Median nerve at wrist	9.9 (2.1) *N* = 15	8.8 (1.1) *N* = 20	11.1 (3.1) *N* = 14	8.3 (2.5) *N* = 8
Median nerve at forearm	6.7 (2.1) *N* = 15	7.3 (2.0) *N* = 20	6.9 (2.0) *N* = 14	5.3 (1.7) *N* = 8
Median nerve at elbow	9.5 (2.4) *N* = 15	11.6 (4.1) *N* = 20	10.9 (2.3) *N* = 14	8.4 (2.0) *N* = 8
Median nerve at arm	10.8 (2.2) *N* = 15	12.5 (4.5) *N* = 20	9.6 (2.4) *N* = 14	8.5 (1.6) *N* = 8
Median nerve at axilla	11.4 (3.3) *N* = 9	13.4 (8.4) *N* = 10	11.6 (3.0) *N* = 14	9.6 (2.5) *N* = 8
Ulnar nerve at wrist	5.3 (1.6) *N* = 15	4.9 (1.1) *N* = 20	5.9 (1.7) *N* = 14	4.8 (1.4) *N* = 8
Ulnar nerve at forearm	6.2 (1.0) *N* = 15	6.5 (1.8) *N* = 20	5.4 (1.3) *N* = 14	5.4 (2.5) *N* = 8
Ulnar nerve at elbow	10.1 (2.6) *N* = 15	10.1 (2.7) *N* = 20	7.9 (2.0) *N* = 14	9.0 (1.3)*N* = 8
Ulnar nerve at arm	7.8 (2.4) *N* = 15	8.6 (2.6) *N* = 20	7.4 (1.9) *N* = 14	7.6 (1.8) *N* = 8
Ulnar nerve at axilla	7.7 (2.4) *N* = 9	7.9 (2.2) *N* = 10	6.6 (2.2) *N* = 14	7.3 (1.3) *N* = 8
Radial nerve at spiral groove	5.9 (1.7) *N* = 15	6.4 (2.3) *N* = 18	5.4 (1.9) *N* = 14	4.5 (1.5) *N* = 8
Posterior interosseus nerve	2.2 (0.7) *N* = 13	2.1 (0.6) *N* = 16	2.4 (0.7) *N* = 13	1.8 (0.5) *N* = 8
Brachial plexus at supraclavicular space	85.6 (8.0) *N* = 8	110.3 (27.9) *N* = 16	103.4 (23.6) *N* = 14	83.3 (17.3) *N* = 8
C5 root	7.3 (3.1) *N* = 10	7.8 (1.5) *N* = 13	6.1 (2.2) *N* = 14	7.4 (3.1) *N* = 8
C6 root	10.1 (3.5) *N* = 10	9.6 (2.1) *N* = 13	9.2 (2.1) *N* = 14	9.4 (2.8) *N* = 8
C7 root	9.9 (3.5) *N* = 9	11.7 (3.6) *N* = 13	10.8 (3.4) *N* = 14	9.9 (1.5) *N* = 8
Fibular nerve at fibular head	10.2 (2.5) *N* = 15	10.0 (3.3) *N* = 18	7.9 (2.5) *N* = 14	9.4 (2.1) *N* = 8
Fibular nerve at popliteal fossa	9.3 (4.1) *N* = 15	7.9 (2.1) *N* = 18	7.1 (2.2) *N* = 14	7.3 (1.0) *N* = 8
Tibial nerve at tarsal tunnel	9.9 (2.7) *N* = 12	11.8 (5.3) *N* = 16	10.0 (2.9) *N* = 14	9.6 (2.9) *N* = 8
Tibial nerve at popliteal fossa	24.9 (6.0) *N* = 13	28.7 (6.5) *N* = 16	23.6 (4.8) *N* = 14	24.1 (3.6) *N* = 8
Sural nerve	3.0 (0.6) *N* = 7	2.1 (0.6) *N* = 10	2.3 (0.5) *N* = 7	2.3 (0.5) *N* = 8
Sciatic nerve at mid-thigh	– *N* = 0	53.3 (10.5) *N* = 6	43.3 (7.7) *N* = 6	38.2 (10.1) *N* = 8

*SD* standard deviation, *PN* polyneuropathy, *N* number of CSA measurements (right and left)

## Discussion

In the present study we performed nerve US evaluation and neurophysiological studies in a consistent group of subjects (both symptomatic patients and pre-symptomatic carriers) with different TTR gene mutations. The results showed that TTR-CTS is characterized by a peculiar mismatch between electrophysiological abnormalities and morphology (ultrasound) of the median nerve at wrist, different from idiopathic CTS, where median nerve CSA mirrors electrophysiology severity regardless of age. To note, the control group (idiopathic CTS) represents the larger population of CTS studied with US so far reported. Secondly, we demonstrated a morphological difference (namely enlarged nerve CSA) of nerve trunks in ATTRv patients with polyneuropathy when compared to pre-symptomatic carriers. Nerve enlargement in ATTRv polyneuropathy is a peculiar feature for an axonal polyneuropathy.

The first result of the present study, the dissociation between morphology (CSA of median nerve at wrist) and neurophysiology of CTS in ATTRv, confirms the findings by Granata et al. [11] in a smaller dataset. One possible explanation for this morpho-functional dissociation may be ascribed to the presence of amyloid deposition in synovial tissue of carpal tunnel structures. Amyloid deposits in the nerves and surrounding ligaments [19] make peripheral nerves more vulnerable to compression injuries. At the compression sites edema, fibrosis, thickened endoneurium, perineurium and the small vessel walls, together with nerve fiber degeneration and regeneration have been described [20]. These factors may interfere with nerve microcirculation leading to ischemic nerve damage. Previous studies in patients with diabetic polyneuropathy and concomitant CTS have shown that the CSA of median nerve was smaller than in diabetic patients without polyneuropathy, thus suggesting the lack of regenerative response to the compression in nerves affected by axonal dysfunction [21, 22]. The last consideration may be applied to ATTRv pre-symptomatic carriers assuming that a pre-clinical axonal dysfunction may precede a still not manifested polyneuropathy. Consistently, an advanced imaging study with MRN [23] showed that nerve ultrastructure is already affected in pre-symptomatic carriers despite the absence of any demonstrable (clinically and neurophysiologically) polyneuropathy. Moreover, TTR has been advocated to play a physiological role in axonal growth and nerve repair mechanisms [24]. Interestingly, in the mouse model of V30M ATTR amyloidosis, nerve regeneration has been shown to be impaired following sciatic nerve injury, due to down-regulation of innate immune responses that play a key role in the tissue regenerative process [25]. It might be speculated that carrying a mutated TTR protein may impair physiological response to compressive nerve injury even before amyloid is deposited. It would be interesting in this respect to evaluate US pattern in patients with CTS due to ATTRwt and immunoglobulin light chain amyloidosis (AL amyloidosis). Previous US evaluations in other hereditary conditions, such as Charcot–Marie–Tooth type 1A and 2, or hereditary neuropathy with liability to pressure palsies (HNPP) do not seem to provide hints for a common interpretation of pathogenetic mechanism [26]. Ginanneschi et al. [27] found enlarged CSA of median nerve at wrist of HNPP patients with CTS, but CSA enlargement was detected also in the majority of HNPP subjects also without CTS [27, 28]. In another study, median nerve at wrist in HNPP was reported to be enlarged in 30% of the examined sample regardless the presence of defined CTS [18]. In axonal CMT (CMT2A) median nerve CSA at wrist showed values ranging between those of controls and CMT1A subjects, but the presence of CTS was not recorded [29]. None of previous studies in hereditary neuropathies focused on the US differences of median nerve with or without CTS or in the US phenotype of mutation carries without an establish hereditary polyneuropathy. Since our study focused on pre-symptomatic carriers with CTS, we cannot compare our results with previous studies because of a different design (no US data on pre-symptomatic HNPP or CMT patients with CTS are reported).

The second result of the present study shows that nerves of ATTRv patients were significantly larger, at US evaluation, than those of pre-symptomatic carriers both at upper and lower limbs, and the increased nerve size was more pronounced proximally (no differences in the distal nerve sites were found in ATTRv patients compared to pre-symptomatic carriers). Interestingly the NIS-LL score correlated with nerve CSA at proximal upper limbs sites (median nerve at axilla, ulnar and radial nerves at arm), to note that proximal sites at lower limbs are not explorable by US. Different physiopathological mechanisms might explain these findings. While the polyneuropathy is, at least at the beginning, distal at limbs, the amyloid deposits are focal and preferentially located at proximal sites [30, 31]. As suggested by pathological findings [30], beside a space-occupying effect of amyloid deposits in the endoneurium, endoneurial edema associated with amyloid deposition in blood vessels and endoneurial interstitium may induce secondary ischemia in nerve fibers, thus causing the progressive polyneuropathy. The co-existence of these mechanisms might explain why an increased nerve CSA (mainly at brachial plexus, according to published CSA values [18, 32, 33]) was measured in an axonal polyneuropathy that is usually associated with CSA values within the normal cut-off [18, 33, 34]. It has also to be considered that CSA measurement of brachial plexus at supraclavicular space may include a large portion of connective tissue than at other sites. However, it is peculiar that we did find these abnormalities only in ATTRv patients with polyneuropathy, and not in the pre-symptomatic carriers, even in the same family (Fig. 2).

Different from the study by Podnar et al. [12], the nerve CSA enlargement in our patients was not strictly limited to the entrapment sites. In the study by Podnar et al. [12], brachial plexus and roots were not evaluated, however CSA of median nerve at axilla was significantly larger in ATTRv patients than in pre-symptomatic carriers, consistent with our findings. It is tempting to speculate that this pattern may reflect a proximal location of amyloid in nerve trunks of patients with polyneuropathy. Moreover, a different pattern of amyloid deposit may depend on the type of TTR mutation.

The present study may suffer from some limitations. Despite a homogeneous and standardized protocol was used, we did not correlate neurophysiological raw data with US findings to minimize potential bias due to the high number of centers involved. Moreover, nerve US was performed in the 7 centers by three different physicians, all with high expertise in the neurosonology of the peripheral nervous system. The study population reflects the Italian ATTRv epidemiology [35] (Italy is a non-endemic country) therefore the clinical findings may be different from those in endemic countries where the prevalence of small fiber neuropathy is higher. Despite the significant differences in nerve CSA in several sites, the range of difference in terms of mm^2^ is too small to be introduced as a potential biomarker for pre-symptomatic carriers except for brachial plexus where the differences are of much larger magnitude. Interpretation of our results remains however speculative.

In conclusion, although the present study missed to identify diagnostic cut-off values, the results may be of high clinical impact. Morpho-functional dissociation in CTS may represent a potential red flag for the diagnosis of ATTRv in patients from non-endemic country with CTS alone, especially in men with bilateral CTS [36]. Tissue analysis might be recommended in these individuals to look for amyloid deposition on flexor tenosynovium when undergoing surgery [37].

In addition, proximal nerve enlargement might be considered a hallmark of ATTRv polyneuropathy able to distinguish the disease from other axonal neuropathies [18, 34]. More important, nerve US monitoring may be considered in the follow-up of the pre-symptomatic carriers with the brachial plexi enlargement being a red flag of diseases occurrence. If these US findings may become a marker of disease progression and/or the response to therapy should be a matter of further studies.

## Data Availability

Data will be shared in anonymous form on a reasoned request to the corresponding author.

## Code availability

Statistical analyses were performed with IBM SPSS Statistic version 23.
